# Supplementary material for: The complete chloroplast genome sequences of six Hylotelephium species: Comparative genomic analysis and phylogenetic relationships
Source: PLoS One. 2023 Oct 10;18(10):e0292056. doi: 10.1371/journal.pone.0292056 (PMC10564136; doi:10.1371/journal.pone.0292056)
Supplement: S3 Table — (DOCX) [file pone.0292056.s004.docx]

**S3 Table. List of genes in the chloroplast genomes of nine *Hylotelephium* species**

| **Function of Genes** | **Group of Genes** | **Gene Names** |
| --- | --- | --- |
| Photosynthesis-related genes | Rubisco | *rbcL* |
|  | PhotosystemⅠ | *psaA, psaB, psaC, psaI, psaJ* |
|  | Assembly and stability of PhotosystemⅠ | *ycf3**, ycf4* |
|  | PhotosystemⅡ | *psbA, psbB, psbC, psbD, psbE, psbF, psbH, psbI, psbJ, psbK, psbL, psbM, psbN, psbT, psbZ* |
|  | ATP synthase | *atpA, atpB, atpE, atpF*, atpH, atpI* |
|  | Cytochrome b/f complex | *petA, petB*, petD*, petG, petL, petN* |
|  | Cytochrome c synthesis | *ccsA* |
|  | NADPH dehydrogenase | *ndhA*, ndhB**(x2)*, ndhC, ndhD, ndhE, ndhF, ndhG, ndhH, ndhI, ndhJ, ndhK* |
| Transcription-and translation-related genes | Transcription | *rpoA, rpoB, rpoC1*, rpoC2* |
|  | Ribosomal proteins | *rpl2**(x2)*, rpl14, rpl16*, rpl20, rpl22, rpl23*(x2)*, rpl32, rpl33, rpl36, rps2, rps3, rps4, rps7*(x2)*, rps8, rps11, rps12**(x2)*, rps14, rps15, rps16*, rps18, rps19* |
| RNA genes | Ribosomal RNA | *rrn4.5S*(x2)*, rrn5S*(x2)*, rrn16S*(x2)*, rrn23S*(x2) |
|  | Transfer RNA | *trnA-UGC**(x2)*, trnC-GCA, trnD-GUC, trnE-UUC, trnF-GAA, trnfM-CAU, trnG-GCC*, trnG-UCC, trnH-GUG, trnI-CAU*(x2)*, trnI-GAU**(x2)*, trnK-UUU*, trnL-CAA*(x2)*, trnL-UAA*, trnL-UAG, trnM-CAU, trnN-GUU*(x2)*, trnP-UGG, trnQ-UUG, trnR-ACG*(x2)*, trnR-UCU, trnS-GCU, trnS-GGA, trnS-UGA, trnT-GGU, trnT-UGU, trnV-GAC*(x2)*, trnV-UAC*, trnW-CCA, trnY-GUA* |
| Other genes | RNA processing | *matK* |
|  | Carbon metabolism | *cemA* |
|  | Fatty acid synthesis | *accD* |
|  | Proteolysis | *clpP*** |
| Gens of unknown function | Conserved reading frames | *ycf1*(x2)*, ycf2*(x2) |

(x2) Duplicated genes; *Gene contains one intron; **Gene contains two introns
