# Supplementary material for: The complete chloroplast genome sequences of six Hylotelephium species: Comparative genomic analysis and phylogenetic relationships
Source: PLoS One. 2023 Oct 10;18(10):e0292056. doi: 10.1371/journal.pone.0292056 (PMC10564136; doi:10.1371/journal.pone.0292056)
Supplement: Attachment — Submitted filename: PONE_reviewers comments.docx [file pone.0292056.s007.docx]

Dear Authors,

Thank you for submitting your manuscript entitled “The complete chloroplast genome sequences of six *Hylotelephium* species: comparative genomic analysis and phylogenetic relationships”. Upon the initial review, I have several comments that will hopefully improve the paper.

1. In the data availability statement, the authors didn’t mention the accession numbers and the database wherein the genomes were deposited.
2. In the paper’s abstract, include a statement or two about the objective of the study.
3. The authors need to establish the ‘necessity’ to sequence the chloroplast genome of *Hylotelephium* species. The introduction part of the paper was not able to establish this clearly due to some confusing and awkward sentence construction. Kindly check the grammar and coherence of the statements. Transition sentences must be included when shifting from one point to another.
4. Please provide a detailed methodology. Include information like the library preparation method and the library insert size. The detailed process of the assembly method should be included in the paper (referenced base or de novo assembly method).
5. The chloroplast genomes’ description should include general details such as the number of duplicated genes, the number of trans-spliced genes, and the GC content of each region (LSC, SSC, IR).
6. It was stated in the paper that the cp genomes of *Hylotelephium* species are highly identical except for the *rps16-trn-UUG*. Did the authors try analyzing the insertions, deletions, and synonymous and non-synonymous substitutions? These might depict the differences among the chloroplast genome sequences.
7. Additional analyses are needed to compare the chloroplast genome sequences (e.g., SNPs analysis, relative codon usage analysis, etc.).
8. The results were simply presented but not explained in the paper, specifically the IR contraction/expansion, repeat analyses, and sequence divergence analysis. The interpretations and implications of the results were not properly discussed. All results presented must be interpreted in the discussion part and the significance of conducting these analyses must be stated in the paper.
9. Supporting details are needed to explain the results or data obtained from the study. The results and discussion should go beyond characterizing the genomes.
10. It was mentioned in the paper that only one region has a significant Pi value. Where did you get the threshold for significance? Is it the same for coding and non-coding regions? It is already established that non-coding regions are highly variable hence when comparing Pi values, it is better to compare the Pi values of the coding regions and non-coding regions separately. In this way, you can check which coding regions have higher nucleotide diversity compared to others.
11. The way the authors described the IR junctions can confuse the readers. Is there any expansion or contraction? What’s the effect of contraction or expansion in the IR junctions?
12. What’s the significance of getting the number of repeats? If one sequence has this number of repeats or repeat types, what’s the implication?
13. Are there any similarities among the *Hylotelephium* species and *Orostachys* species that grouped together in one clade? Or any possible reasons behind the clustering of some *Hylotelephium* species and *Orostachys* species?
14. The figures have low resolution, the details are not visible.
