# Supplementary material for: The complete chloroplast genome sequences of six Hylotelephium species: Comparative genomic analysis and phylogenetic relationships
Source: PLoS One. 2023 Oct 10;18(10):e0292056. doi: 10.1371/journal.pone.0292056 (PMC10564136; doi:10.1371/journal.pone.0292056)
Supplement: Attachment — Submitted filename: PONE-D-22-30339-reviever response.docx [file pone.0292056.s008.docx]

In this manuscript, authors reported six chloroplast genomes of *Hylotelephium* species and compared thirteen chloroplast genomes from *Hylotelephium* and *Orostachys* species published in previous studies. Authors have done detail comparison and characterization of the nineteen chloroplast genomes. The study could be interesting for publication in PLOS ONE. However, the manuscript does have several issues which I think need to be addressed.

Major：
1. The aim (2) to clarify the taxonomic identities of several taxa with ambiguous taxonomic identities. It seems there is no clear answer to this question in manuscript.

2. The aim (3) to provide important information about the most suitable chloroplast molecular markers for further studies to solve unclear phylogenetic relationships of *Hylotelephium.* There is no clear answer to this question in manuscript.
3. The English writing of this mauscript should be improved.

Minor：

1. Fig.1 should list the six *Hylotelephium* species names. The resolution of Fig. 1 was low.
2. Fig. 4 was not clear.
3. Fig. 5 was not clear.
