# Supplementary material for: The complete chloroplast genome sequences of six Hylotelephium species: Comparative genomic analysis and phylogenetic relationships
Source: PLoS One. 2023 Oct 10;18(10):e0292056. doi: 10.1371/journal.pone.0292056 (PMC10564136; doi:10.1371/journal.pone.0292056)
Supplement: Attachment — Submitted filename: Response to Reviewers.docx [file pone.0292056.s009.docx]

**Response to Reviewers**

We are pleased to resubmit for publication the revised version of PONE-D-22-30339 “**The complete chloroplast genome sequences of six *Hylotelephium* species: comparative genomic analysis and phylogenetic relationships**” We appreciated the constructive criticisms of the reviewers. We have addressed each of their concerns as outlined below.

**Journal requirements:**

**1. Please ensure that your manuscript meets PLOS ONE's style requirements, including those for file naming. The PLOS ONE style templates can be found at**

https://journals.plos.org/plosone/s/file?id=wjVg/PLOSOne_formatting_sample_main_body.pdf and

https://journals.plos.org/plosone/s/file?id=ba62/PLOSOne_formatting_sample_title_authors_affiliations.pdf

*→ We checked and reflected the template style of PLOS ONE at the request of the journal.*

**2. We note that you have stated that you will provide repository information for your data at acceptance. Should your manuscript be accepted for publication, we will hold it until you provide the relevant accession numbers or DOIs necessary to access your data. If you wish to make changes to your Data Availability statement, please describe these changes in your cover letter and we will update your Data Availability statement to reflect the information you provide.**

*→ We have added the data availability statement in the manuscript.*

**Reviewer 1.**

**Chloroplast is a maternal genetic marker. Plant classification is mainly based on morphological characteristics, which is mostly consistent with the phylogenetic results of nuclear genetic markers inherited by parents. In plants, the phylogenetic results of chloroplast genetic markers and nuclear genetic markers are often different. Therefore, the introduction of the author in the introduction and discussion should clearly state which kind of marker the previous research results are based on. Moreover, based on the above reasons, the results of this study are not enough to determine the systematic location of *H. viridescens*, but it does provide useful information. The author may supplement the phylogenetic results of nuclear genetic markers, or please explain carefully in the results and discussion section.**

*→ Thank you very much for your constructive comments.*

*→ We fully agree your opinion what ‘plant classification is mainly based on morphological characteristics, which is mostly consistent with the phylogenetic results of nuclear markers inherited by parents’.*

*→ We have revised the Introduction and Discussion sections based on what you pointed out. Thank you again.*

**Reviewer 2.**

**Thank you for submitting your manuscript entitled “The complete chloroplast genome sequences of six *Hylotelephium* species: comparative genomic analysis and phylogenetic relationships”. Upon the initial review, I have several comments that will hopefully improve the paper.**

1. In the data availability statement, the authors didn’t mention the accession numbers and the database wherein the genomes were deposited.

*→* We have deposited the genome sequences of six *Hylotelephium* species in the GenBank of NCBI and provided the GenBank accession numbers in Table 1 and S1 Table.

2. In the paper’s abstract, include a statement or two about the objective of the study.

*→ We have added it in the Abstract section.*

3. The authors need to establish the ‘necessity’ to sequence the chloroplast genome of Hylotelephium species. The introduction part of the paper was not able to establish this clearly due to some confusing and awkward sentence construction. Kindly check the grammar and coherence of the statements. Transition sentences must be included when shifting from one point to another.

*→ We have revised the Introduction section to emphasize the necessity of this study.*

4. Please provide a detailed methodology. Include information like the library preparation method and the library insert size. The detailed process of the assembly method should be included in the paper (referenced base or de novo assembly method).

*→ We have added and revised it.*

5. The chloroplast genomes’ description should include general details such as the number of duplicated genes, the number of trans-spliced genes, and the GC content of each region (LSC, SSC, IR).

*→ We have added it.*

6. It was stated in the paper that the cp genomes of Hylotelephium species are highly identical except for the rps16-trn-UUG. Did the authors try analyzing the insertions, deletions, and synonymous and non-synonymous substitutions? These might depict the differences among the chloroplast genome sequences.

*→ It is only a description of mVISTA analysis results. Also, we carried out the sequence comparison analyses in sequence divergence and mutational hotspots section of this paper.*

7. Additional analyses are needed to compare the chloroplast genome sequences (e.g., SNPs analysis, relative codon usage analysis, etc.).

*→ We have added the relative codon usage analysis.*

8. The results were simply presented but not explained in the paper, specifically the IR contraction/expansion, repeat analyses, and sequence divergence analysis. The interpretations and implications of the results were not properly discussed. All results presented must be interpreted in the discussion part and the significance of conducting these analyses must be stated in the paper.

*→ We have added some sentences.*

9. Supporting details are needed to explain the results or data obtained from the study. The results and discussion should go beyond characterizing the genomes.

*→ We fully agree your opinion and have done my best to do so.*

10. It was mentioned in the paper that only one region has a significant Pi value. Where did you get the threshold for significance? Is it the same for coding and non-coding regions? It is already established that non-coding regions are highly variable hence when comparing Pi values, it is better to compare the Pi values of the coding regions and non-coding regions separately. In this way, you can check which coding regions have higher nucleotide diversity compared to others.

*→ It means relatively high, and we have added the word ‘relatively’ to the manuscript*

*→ Also, what you said is a very good method, but we think the sliding window method is also a good method. In particular, we think that the biggest advantage of sliding window method is that it directly selects marker regions suitable for future phylogenetic studies by analyzing the length that can be analyzed by Sanger sequencing. because of this advantage, we analyzed using the sliding window method in this study.*

*→ We ask for your kind understanding.*

11. The way the authors described the IR junctions can confuse the readers. Is there any expansion or contraction? What’s the effect of contraction or expansion in the IR junctions?

*→ We have revised it.*

12. What’s the significance of getting the number of repeats? If one sequence has this number of repeats or repeat types, what’s the implication?

*→ Repeat sequences are considered important information in population genetics. So we analyzed SSR to provide information for future population genetics studies of Hylotelephium. Also, we have added it in the manuscript.*

13. Are there any similarities among the Hylotelephium species and Orostachys species that grouped together in one clade? Or any possible reasons behind the clustering of some Hylotelephium species and Orostachys species?

*→ We think it is because of the limitation of the resolution of the cp genome in this group. In our next paper, which is being prepared based on single copy gene sequences of nuclear genome, each section of Hylotelephium and Orostachys formed independent monophyletic group and were perfectly separated.*

14. The figures have low resolution, the details are not visible.

*→ We have modified the resolution of all figures to 300 dpi.*

**Reviewer 3.**

In this manuscript, authors reported six chloroplast genomes of Hylotelephium species and compared thirteen chloroplast genomes from Hylotelephium and Orostachys species published in previous studies. Authors have done detail comparison and characterization of the nineteen chloroplast genomes. The study could be interesting for publication in PLOS ONE. However, the manuscript does have several issues which I think need to be addressed.

Major：

1. The aim (2) to clarify the taxonomic identities of several taxa with ambiguous taxonomic identities. It seems there is no clear answer to this question in manuscript.

*→ We have revised the aim (2).*

2.The aim (3) to provide important information about the most suitable chloroplast molecular markers for further studies to solve unclear phylogenetic relationships of Hylotelephium. There is no clear answer to this question in manuscript.

*→ We have revised the aim (3).*

3.The English writing of this mauscript should be improved.

*→ Manuscript has been completely revised by a professional English translation agency.*

Minor：

1.Fig.1 should list the six Hylotelephium species names. The resolution of Fig. 1 was low.

2.Fig. 4 was not clear.

3.Fig. 5 was not clear.

*→ We have modified the resolution of all figures to 300 dpi and modified Figures 4 and 5.*
