# Supplementary material for: The complete chloroplast genome sequences of six Hylotelephium species: Comparative genomic analysis and phylogenetic relationships
Source: PLoS One. 2023 Oct 10;18(10):e0292056. doi: 10.1371/journal.pone.0292056 (PMC10564136; doi:10.1371/journal.pone.0292056)
Supplement: Attachment — Submitted filename: PONE-D-22-30339-reviever response2.docx [file pone.0292056.s010.docx]

In this manuscript, authors reported six chloroplast genomes of *Hylotelephium* species and compared thirteen chloroplast genomes from *Hylotelephium* and *Orostachys* species published in previous studies. Authors have done detail comparison and characterization of the nineteen chloroplast genomes. The manuscript has improved. I agree to accept for publication in PLOS ONE.
