# Supplementary material for: The complete chloroplast genome sequences of six Hylotelephium species: Comparative genomic analysis and phylogenetic relationships
Source: PLoS One. 2023 Oct 10;18(10):e0292056. doi: 10.1371/journal.pone.0292056 (PMC10564136; doi:10.1371/journal.pone.0292056)
Supplement: Attachment — Submitted filename: Comments.docx [file pone.0292056.s011.docx]

Thank you for submitting the revised manuscript. I appreciate the efforts in addressing the reviewers’ comments, but upon reading the revision, I have additional comments you might consider in improving the paper.

1. The introductory parts need refinement. The description of the genus must be rephrased to fit in the paragraph, the way its phrase right now seems awkward. An awkwardly written statement can be technically correct but may sound “rough” and not proofread.
2. The third paragraph shows the disagreement on the taxonomy of *Hylotelephium* but the reasons behind the disagreement or differences in opinions were not properly discussed. The paragraph mentioned that “Gray [9] recognized this genus as a section of *Sedum*, and many taxonomists [2,10-12] agreed with Gray’s opinions, but *Clausen* [13] disagreed with this and classified it as a subgenus of Sedum” but there’s no follow-up statement on the reasons behind these differences in taxonomic views. What could be the morphological bases of their claims?
3. In the methodology part, kindly include the library insert size and library preparation method.
4. The result of mVista alignment needs further discussion. The statement “The LSC and SSC regions were more variable than the IR regions” does not fully interpret the result.
5. The implications of the following results are still unclear in the discussion part. How can you explain the following results?
   a. High GC content in the IR regions
   b. reverse and complement repeats were only present only in *H. ewersii* (1 repeat) and *H. pallescens* (2 repeats), respectively
   c. Mononucleotide repeats were the most abundant, with A/T repeats being the only represented repeats
6. As indicated in the discussion part “Most of the sequences of the intergenic spacer between *rps*16-*trn*Q(UUG) were deleted. It is considered a relatively large event in the highly conserved *Hylotelephium* chloroplast genome”. But if deletion events are relatively important in sequence variation analysis, why not consider performing additional analysis that will highlight the mutational hotspots or nucleotide substitutions? Analysis of the SNPs and Indels or even the ratio of nonsynonymous to synonymous nucleotide substitution rates (Ka/Ks) might give more information about the sequence divergence. For instance, the Ka/Ks ratio is used to divide genes into positive selection, neutral evolution, and purification, with a limit of one. The genes with the highest Ka/Ks variability can be used as candidate barcodes to differentiate species and in the future applied to perform phylogenetic and phylogeographic analyses.
7. In the phylogeny part, it was indicated that “We do not believe these results can be interpreted as a conclusion that the two genera should be combined into one genus because the morphological characteristics between the two genera greatly differ”, can you elaborate on this?
8. In general, the paper can be enhanced by providing an impactful and effective discussion of results. The discussion must inform readers about the larger implications of your study based on the results.
