# Supplementary material for: The complete chloroplast genome sequences of six Hylotelephium species: Comparative genomic analysis and phylogenetic relationships
Source: PLoS One. 2023 Oct 10;18(10):e0292056. doi: 10.1371/journal.pone.0292056 (PMC10564136; doi:10.1371/journal.pone.0292056)
Supplement: Attachment — Submitted filename: Response to Reviewers.docx [file pone.0292056.s012.docx]

**Response to Reviewers**

We are pleased to resubmit for publication the revised version of PONE-D-22-30339 “**The complete chloroplast genome sequences of six *Hylotelephium* species: comparative genomic analysis and phylogenetic relationships**” We appreciated the constructive criticisms of the reviewers. We have addressed each of their concerns as outlined below.

**Reviewer 1.**

No Response

*→ Thank you very much for agreeing to publish our paper in PLOS ONE.*

**Reviewer 2.**

Thank you for submitting the revised manuscript. I appreciate the efforts in addressing the reviewers’ comments, but upon reading the revision, I have additional comments you might consider in improving the paper.

1. The introductory parts need refinement. The description of the genus must be rephrased to fit in the paragraph, the way its phrase right now seems awkward. An awkwardly written statement can be technically correct but may sound “rough” and not proofread.

*→ We have revised it.*

2. The third paragraph shows the disagreement on the taxonomy of *Hylotelephium* but the reasons behind the disagreement or differences in opinions were not properly discussed. The paragraph mentioned that “Gray [9] recognized this genus as a section of *Sedum*, and many taxonomists [2,10-12] agreed with Gray’s opinions, but *Clausen* [13] disagreed with this and classified it as a subgenus of Sedum” but there’s no follow-up statement on the reasons behind these differences in taxonomic views. What could be the morphological bases of their claims?

*→ Unfortunately, many older taxonomic literatures, including those mentioned above, did not explained why. So, we couldn’t write about the reason either.*

3. In the methodology part, kindly include the library insert size and library preparation method.

*→ We have added it.*

4. The result of mVista alignment needs further discussion. The statement “The LSC and SSC regions were more variable than the IR regions” does not fully interpret the result.

*→ We have revised and added it.*

5. The implications of the following results are still unclear in the discussion part. How can you explain the following results?
   a. High GC content in the IR regions
   b. reverse and complement repeats were only present only in *H. ewersii* (1 repeat) and *H. pallescens* (2 repeats), respectively
   c. Mononucleotide repeats were the most abundant, with A/T repeats being the only represented repeats

*→ They are general descriptions of the results of the analysis of cp genome of this group.*

*→ It would be nice if all the results were connected to the discussion, but we think there are times when it is not necessary or not possible to do so.*

6. As indicated in the discussion part “Most of the sequences of the intergenic spacer between *rps*16-*trn*Q(UUG) were deleted. It is considered a relatively large event in the highly conserved *Hylotelephium* chloroplast genome”. But if deletion events are relatively important in sequence variation analysis, why not consider performing additional analysis that will highlight the mutational hotspots or nucleotide substitutions? Analysis of the SNPs and Indels or even the ratio of nonsynonymous to synonymous nucleotide substitution rates (Ka/Ks) might give more information about the sequence divergence. For instance, the Ka/Ks ratio is used to divide genes into positive selection, neutral evolution, and purification, with a limit of one. The genes with the highest Ka/Ks variability can be used as candidate barcodes to differentiate species and in the future applied to perform phylogenetic and phylogeographic analyses.

*→ We fully agree your opinion.*

*→ However, we believe that we have performed analyzes and discussions that sufficiently explain of our study aim.*

7. In the phylogeny part, it was indicated that “We do not believe these results can be interpreted as a conclusion that the two genera should be combined into one genus because the morphological characteristics between the two genera greatly differ”, can you elaborate on this?

*→ We have added it.*

8. In general, the paper can be enhanced by providing an impactful and effective discussion of results. The discussion must inform readers about the larger implications of your study based on the results.

*→ Thank you very much for your sincere advice.*

**Reviewer 3.**

In this manuscript, authors reported six chloroplast genomes of Hylotelephium species and compared thirteen chloroplast genomes from *Hylotelephium* and *Orostachys* species published in previous studies. Authors have done detail comparison and characterization of the nineteen chloroplast genomes. The manuscript has improved many areas, such as figures solutions and English writing. I agree to accept for publication in PLOS ONE.

*→ Thank you very much for your kind comments and for agreeing to publish our paper in PLOS ONE.*
